# Learning Curve for Left Bundle Branch Area Pacing Lead Implantation

**DOI:** 10.19102/icrm.2025.16055

**Published:** 2025-05-15

**Authors:** Maci Clark, Hannah Zerr, Ben Ose, David Fritz, Caroline Trupp, Amulya Gupta, Ahmed Shahab, Amit Noheria, Seth H. Sheldon

**Affiliations:** 1 The University of Kansas School of Medicine, Kansas City, KS, USA; 2 Department of Cardiovascular Medicine, The University of Kansas Medical Center, Kansas City, KS, USA; 3 Department of Cardiovascular Medicine, Southern Illinois University School of Medicine, Springfield, IL, USA

**Keywords:** Complications, conduction system pacing, learning curve, left bundle branch area pacing, operator experience

## Abstract

Left bundle branch area pacing (LBBAP) has shown promising outcomes at experienced centers; however, less is known about the learning curve with initial adoption of LBBAP implantation. We conducted a retrospective analysis (2020–2023) of the learning curve for LBBAP at an academic medical center. Procedural success and device-related adverse events in adult patients undergoing LBBAP by seven new operators with >5 years’ experience in device implantation were compared between operators with a history of ≤10 (LBBAP_inexp_) versus >10 (LBBAP_exp_) LBBAP implant attempts. Successful LBBAP was defined as a left ventricular activation time (LVAT) of ≤80 ms. Seven operators implanted LBBAP devices in 288 patients (age, 73 ± 11 years; 38% women), including 68 (24%) in the LBBAP_inexp_ group versus 220 (76%) patients in the LBBAP_exp_ group with similar baseline characteristics. The median number of implants per operator was 22 (range, 8–83). Post-implant LVAT ≤ 80 ms was less frequent in LBBAP_inexp_ compared to LBBAP_exp_ (56.9% vs 72.4%; *P* = .04). There were no significant differences in paced QRS duration ≤ 130 ms (75.9% vs. 76.1%; *P* = 1.0) or operator self-identified success (85% vs. 91%; *P* = .2). With new single-/dual-chamber device implants, there was no difference in implant duration (103.4 ± 31.8 vs. 101.6 ± 38.5 min; *P* = .3), but there was longer fluoroscopy with LBBAP_inexp_ (12.6 ± 10.1 vs. 8.2 ± 8.0 min; *P* < .0001). The average number of attempts at LBBAP was lower with LBBAP_inexp_ versus LBBAP_exp_ (2.0 ± 1.5 vs. 2.9 ± 2.9; *P* = .03). There was no difference in device-related adverse events between the two groups (*P* = .3). Operators use less fluoroscopy, make more attempts at LBBAP, and more frequently achieve LVAT ≤ 80 ms after their first 10 implants.

## Introduction

Left bundle branch area pacing (LBBAP) presents an alternative to conventional methods of right ventricular pacing (RVP) and cardiac resynchronization therapy in patients undergoing pacemaker implantation. Conventional RVP uses lead placement in the right ventricle, which results in dyssynchronous left ventricular (LV) activation resembling a left bundle branch block pattern. Dyssynchronous ventricular contraction can contribute to heart failure (HF), increasing the risk of HF-related hospitalization and death.^1,2^ Historically, biventricular pacing (BiVP) involving the addition of an LV lead via the coronary sinus has been used in patients with established systolic HF and dyssynchronous LV activation to improve interventricular synchrony.^3^ However, BiVP can be difficult to accomplish in the setting of challenging coronary sinus anatomy, lateral LV scar, and phrenic nerve stimulation. Additionally, around one-third of BiVP patients have a poor response after implantation due in part to lead positioning issues, lead instability, and suboptimal device programming.^4,5^

Conduction system pacing, which includes His-bundle pacing (HBP) and LBBAP, is a ventricular pacing strategy to preserve electrical and mechanical LV synchrony via direct activation of the conduction system. Conduction system pacing is more physiologic than BiVP, results in a narrower paced QRS complex, and is often a simpler solution without the requirement of an additional lead to be implanted in the coronary venous system.^6^

LBBAP involves placing the pacemaker lead directly into the interventricular septum in proximity to the left bundle branch (LBB) to allow for a more synchronized ventricular activation pattern due to recruitment of the conduction system. LBBAP is associated with better chronic stability of the pacing lead with a lower and more stable pacing capture threshold as compared to HBP or BiVP.^7–9^ At most centers, LBBAP is preferred over HBP due to the former having an easier and more successful implant procedure with a shorter procedure duration and better long-term lead reliability.^10^

A successful LBBAP implantation can be determined by specific electrocardiographic (ECG) findings, including a narrowed QRS complex and an increased R-wave amplitude in lead V1.^9,11^ In previous studies, LBBAP has been shown to have similar success rates to RVP but requires a long learning curve.^12^ While LBBAP is not yet adapted as the standard of treatment across the United States, it is important to assess the learning curve with adoption of LBBAP. We hypothesize that LBBAP success is higher with increased operator experience. In this study, we sought to evaluate the learning curve for LBBAP implantation during its initial adoption at our academic center.

## Methods

### Study population

This single-center retrospective cohort study included adult patients (≥18 years of age) who underwent an LBBAP lead implantation attempt at The University of Kansas Medical Center between September 2020 and January 2023. Procedures included both first-choice LBBAP lead implantations and rescue LBBAP lead implantations in cases of failed attempts at other lead locations. Cases where LBBAP was not attempted were not included. His-bundle lead implants were excluded.

### Left bundle branch area pacing procedural success

Outcome measures of LBBAP procedural success included the following:

1. A pacing stimulus to peak of R-wave in lead V6, also known as the LV activation time (LVAT), of ≤80 ms on a 12-lead ECG recorded the day after device implant;
2. A paced QRS duration (QRSd) of ≤130 ms on a 12-lead ECG recorded the day after device implant; and
3. The implanting operator’s subjective confirmation of a “successful” LBBAP implant

### Baseline clinical and procedural variables

Baseline clinical characteristics were recorded from electronic medical records.

All primary operators who performed LBBAP implantations had ≥5 years’ experience with conventional pacemaker implantation. There was no standard training the operators were required to undergo before starting LBBAP implants. Most operators had some experience with HBP and learned the LBBAP technique from previously published literature, available online resources, and from each other/external peers.

Procedural characteristics were collected from the post-procedure operative report. The variables collected included total procedure time (min), total fluoroscopy time (min), start-to-finish time from first incision (min), and operator details. The start-to-finish and fluoroscopy times were limited to new single- and dual-chamber device implantations, as these variables are assumed to be more variable and reflective of complexities outside of LBBAP lead implantation, with more complex procedures including extractions and/or upgrades. Additionally, we obtained the number of LBBA lead implant attempts as described in the procedure report.

### Electrocardiography, adverse events, and outcomes

Results were collected from ECGs recorded on the day after the implant procedure. ECGs were obtained during forced ventricular pacing in the VVI mode predominantly with a 2-V output at 0.4 ms during device interrogation. ECG variables evaluated for this analysis included LVAT and QRSd.

Device-related adverse events were reviewed in the initial 30 days post-procedure. They included implant-related deep vein thrombosis, device infection, any lead microperforation needing revision, pericardial effusion or tamponade requiring intervention, pneumothorax, or pocket hematoma. We separately report lead-related adverse events; these included lead failure, which was defined as any documented instance of lead malfunction, including lead fractures and major electrical abnormalities such as a markedly elevated threshold. These events were distinguished from lead dislodgements, which were recorded separately. Myocardial infarction, stroke/transient ischemic attack (TIA), and all-cause mortality within 30 days of implant were also recorded after the LBBAP procedure.

### Statistical analysis

Non-identifiable patient data were stored electronically using Research Electronic Data Capture (REDCap). The data were split into two groups based on the number of LBBAP implant procedures to compare success rates between operators and gained experience. Based on results from the previous literature, LBBAP implants were stratified by the initial 10 attempted implantation procedures per operator (LBBAP_inexp_) and their subsequent LBBAP procedures (LBBAP_exp_).^13^ Continuous variables are expressed as mean ± standard deviation values, while categorical variables are expressed as frequencies and percentages. For comparisons of continuous variables, we used Student’s independent-sample *t* test (except for very skewed variables, for which we used the non-parametric Wilcoxon rank-sum test). For comparisons of categorical variables, Fisher’s exact test was used. We considered a two-tailed *P* value of ≤.05 to indicate statistical significance. Analyses were conducted using JMP Pro version 17 (SAS Institute, Cary, NC, USA).

## Results

**Figure 1:**
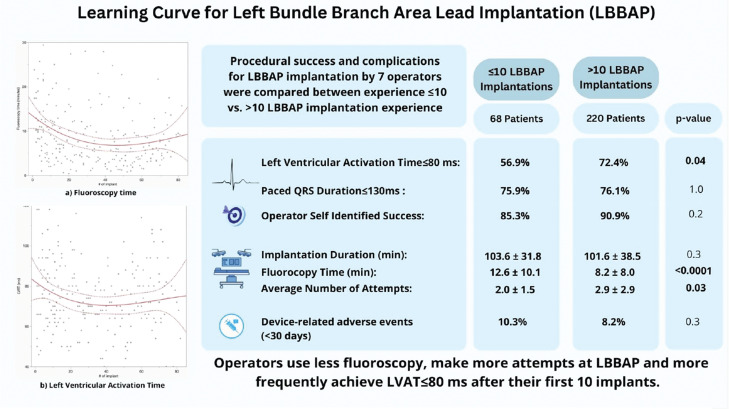
Summary of findings for the learning curve of left bundle branch area pacing lead implantation.

The results of this study are summarized in **Figure 1**.

### Baseline characteristics of left bundle branch area pacing patients

A total of 288 patients (age, 73 ± 11 years; 38% women) were included in the study who underwent an LBBAP lead implantation attempt. There were 68 patients in the LBBAP_inexp_ group and 220 patients in the LBBAP_exp_ group. Most of the population was white/Caucasian (85.3%) with an elevated body mass index (29.9 ± 6.7 kg/m^2^), with no significant differences between groups **(Table 1)**. The majority of patients had attempted LBBAP lead implantation with a 3830 lead (Medtronic, Minneapolis, MN, USA) (n = 265, 92.0%). The remainder had attempted LBBAP with Ingevity+ leads (n = 20, 6.9%) (Boston Scientific, Marlborough, MA, USA), Solia leads (n = 2, 0.7%) (Biotronik, Berlin, Germany), or the Boston Scientific Fineline II 4471 lead (n = 1, 0.4%). There were no differences in chronic health conditions at baseline between the two groups, and the average LV ejection fraction was 56% ± 11%.

**Table 1:** Baseline Characteristics

Variable	Overall (n = 288)	≤10 LBBAP Cases (n = 68)	>10 LBBAP Cases (n = 220)	*P* Value
Age, years	73.3 ± 10.7	74.8 ± 10.5	72.9 ± 10.8	.2
Female sex	108 (37.5%)	25 (36.8%)	83 (37.7%)	.9
Race/ethnicity				
White	246 (85.3%)	58 (84.9%)	188 (85.5%)	.3
Black or AA	24 (8.3%)	4 (6.1%)	20 (8.9%)	
Hispanic	12 (4.2%)	3 (4.6%)	9 (4.1%)	1.0
BMI, kg/m^2^	29.9 ± 6.7	29.9 ± 7.2	29.9 ± 6.6	1.0
Comorbidities				
Hypertension	227 (78.8%)	59 (86.8%)	168 (76.4%)	.09
Diabetes mellitus	98 (34.0%)	26 (38.2%)	72 (32.7%)	.4
Chronic obstructive pulmonary disease	47 (16.3%)	11 (16.2%)	36 (16.3%)	1.0
Obstructive sleep apnea	87 (30.2%)	21 (30.9%)	66 (30.0%)	.9
End-stage renal disease	6 (2.1%)	1 (1.5%)	5 (2.3%)	1.0
Coronary artery disease	139 (48.2%)	34 (50%)	105 (47.8%)	.8
History of atrial fibrillation	132 (45.8%)	30 (44.1%)	102 (46.3%)	.8
Congestive heart failure	26 (9.0%)	7 (10.5%)	19 (8.8%)	.6
Left ventricular ejection fraction, %	56 ± 11	55 ± 10	56 ± 12	.5

*Abbreviations:* AA, African American; BMI, body mass index; LBBAP, left bundle branch area pacing.

### Learning curve and success rates

There were seven different operators included in the study, with varying numbers of LBBAP implants. The median number of implants per operator was 22 (range, 8–83).

Successful LBBAP lead implantation, as defined by LVAT ≤ 80 ms, was significantly lower in the LBBAP_inexp_ group compared to the LBBAP_exp_ group (56.9% vs. 72.4%; *P* = .04). This difference remained significant with adjustment for the operator performing the procedure (*P* = .03). There was no difference in the frequency of paced QRSd ≤ 130 ms between the LBBAP_inexp_ and LBBAP_exp_ groups (75.9% vs. 76.1 %; *P* = 1.0). Successful LBBAP lead implantation as self-defined by the implanter was similar between the LBBAP_inexp_ and LBBAP_exp_ groups (85% vs. 91%; *P* = .2).

**Figure 2:**
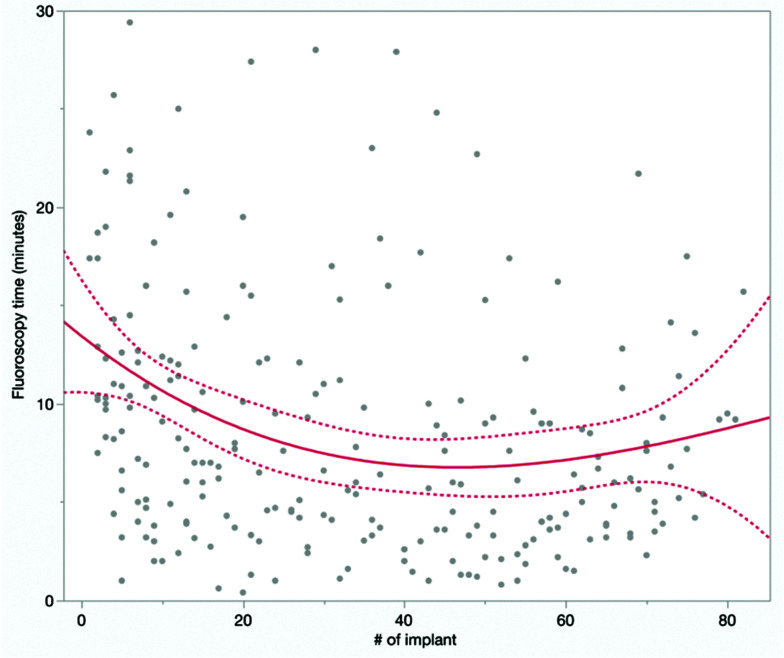
Fluoroscopy time in minutes for new single-/dual-chamber device implants with attempted left bundle branch area pacing plotted against the chronological number of implants for the operator. The best-fit cubic spline with 95% confidence intervals is shown.

In new single- or dual-chamber implants, there was no difference in implant duration, but there was a significant difference in the reduction in fluoroscopy time between the LBBAP_inexp_ and LBBAP_exp_ groups (**Table 2**; 12.6 ± 10.1 vs. 8.2 ± 8.0 min; *P* < .0001). **Figure 2** shows the plot of fluoroscopy time against experience (number of implants) with a fitted cubic spline. The number of attempts at LBBAP was less in the LBBAP_inexp_ group than in the LBBAP_exp_ group (2.0 ± 1.5 attempts in LBBAP_inexp_ vs. 2.9 ± 2.9 attempts in LBBAP_exp_; *P* = .03).

**Table 2:** Comparison Between Procedural and Electrocardiogram Variables by Left Bundle Branch Area Pacing Implantation Experience

Variable	Overall			Successful Procedures (Self-determined by Operator)		
	≤10 LBBAP Cases (n = 68)	>10 LBBAP Cases (n = 220)	*P* Value	≤10 LBBAP Cases (n = 58)	>10 LBBAP Cases (n = 200)	*P* Value
Procedure variables						
Successful LBBAP determined by operator	58 (85.3%)	200 (90.9%)	.2	—	—	
Implant duration (min)^a^	103.6 ± 31.8	101.6 ± 38.5	.3^b^	99.9 ± 30.3	97.2 ± 33.6	.3^b^
Fluoroscopy time (min)^a^	12.6 ± 10.1	8.2 ± 8.0	<.0001*^b^	12.3 ± 10.1	7.3 ± 5.8	<.0001*^b^
Number of LBBAP implant attempts	2.0 ± 1.5	2.9 ± 2.9	.03*^b^	1.9 ± 1.5	2.7 ± 2.3	.01*^b^
LV perforation	3 (4.4%)	18 (8.2%)	.4	2 (3.5%)	18 (9.0%)	.3
Post-implant ECG^c^						
Paced QRS duration, ms^d^	117.8 ± 22.4	119.3 ± 20.0	.7	115.4 ± 22.0	117.4 ± 17.9	.8
Paced QRS duration ≤130 ms^d^	41 (75.9%)	143 (76.1%)	1.0	40 (81.6%)	142 (78.9%)	.8
LVAT, ms^d^	78.6 ± 21.3	72.4 ± 19.6	.07	78.0 ± 20.9	72.1 ± 19.3	.09
LVAT ≤80 ms	29 (56.9%)	134 (72.4%)	.04*	27 (58.7%)	131 (73.2%)	.07

*Abbreviations:* ECG, electrocardiography; LBBAP, left bundle branch area pacing; LV, left ventricular; LVAT, left ventricular activation time. ^a^Limited to new single- or dual-chamber device implantation. ^b^Non-parametric test. ^c^Excluding missing values. ^d^Minimum of bipolar and unipolar when both available. *Statistically significant.

### Post-implant electrocardiographic data

**Figure 3:**
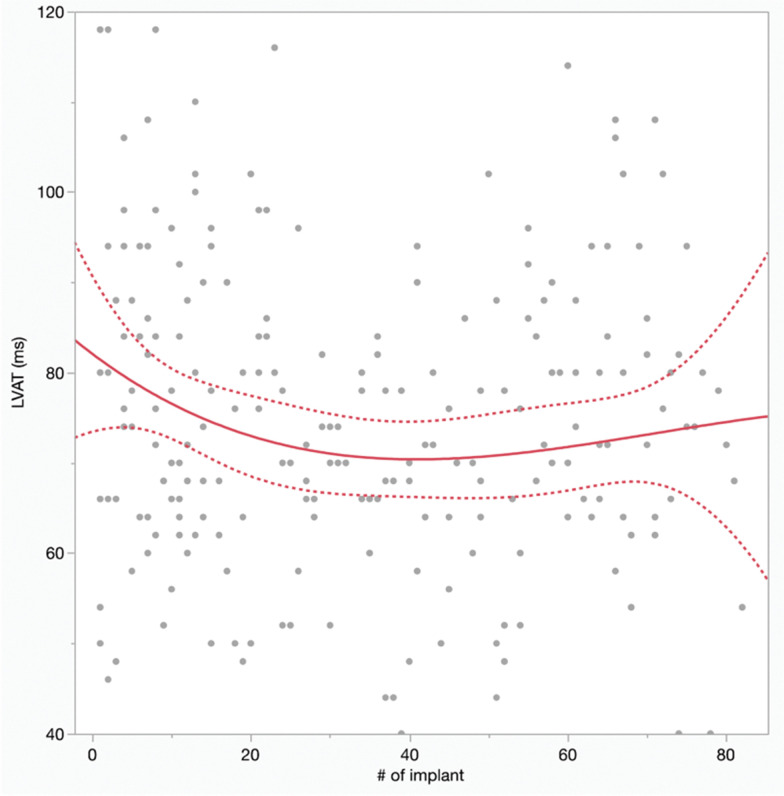
Ventricular pacing left ventricular activation time (ms) for device implants with attempted left bundle branch area pacing plotted against the chronological number of implants for the operator. The best-fit cubic spline with 95% confidence intervals is shown.

There were no significant differences in the ventricular paced QRSd for the LBBAP_inexp_ versus LBBAP_exp_ groups (117.8 ± 22.4 vs. 119.3 ± 20.0 ms; *P* = .7). An LVAT ≤ 80 ms was achieved in fewer LBBAP_inexp_ cases compared to LBBAP_exp_ cases (as described earlier), and there was a trend toward a longer LVAT in the LBBAP_inexp_ group versus the LBBAP_exp_ group (**Table 2**; 78.6 ± 21.3 vs. 72.4 ± 19.6 ms; *P* = .07). **Figure 3** shows the plot of LVAT against experience (number of implants) with a cubic spline to fit the data.

### Device-related adverse events

Overall, there were 25 (8.7%) device-related adverse events within 30 days of device implantation **(Table 3)**. The adverse event rates were similar between the LBBAP_inexp_ and LBBAP_exp_ groups (10.3% vs. 8.2%; *P* = .3). The most common adverse event was LBBAP lead dislodgement, with the LBBAP_inexp_ group having two (2.9%) dislodgements, while the LBBAP_exp_ group had four (1.8%). There was no statistical difference between the two groups (*P* = .6). Among the three (1.1%) patients with pericardial effusion/tamponade, two underwent atrial lead revision alone and the third did not require lead intervention (they had decompensated HF with pleural and pericardial effusions). Device infection occurred in one patient who was in the LBBAP_exp_ group (0.5%) and later underwent device extraction. There were no myocardial infarctions or stroke/TIA within 30 days in either group. There were two (2.9%) all-cause deaths within 30 days in the LBBAP_inexp_ group and zero (0%) all-cause deaths in the LBBAP_exp_ group (*P* = .06). None of the deaths were due to implant-related causes. There was no significant difference in any specific adverse event between the groups.

**Table 3:** Comparison of 30-day Adverse Events by Left Bundle Branch Area Pacing Implantation Experience

Variable	Overall (n = 288)	≤10 LBBAP Cases (n = 68)	>10 LBBAP Cases (n = 220)	*P* Value
Total device-related adverse events (≤30 days)	25 (8.7%)	7 (10.3%)	18 (8.2%)	.3
Procedure-related complications	13 (4.5%)	2 (2.9%)	11 (5.0%)	.5
Implant-related DVT	1 (0.3%)	0 (0%)	1 (0.5%)	1.0
Device infection	1 (0.3%)	0 (0%)	1 (0.5%)	1.0
Any lead microperforation resulting in revision	2 (0.7%)	0 (0%)	2 (0.9%)	.4
Pericardial effusion/tamponade requiring intervention	3 (1.1%)	0 (0%)	3 (1.4%)	1.0
Pneumothorax	4 (1.4%)	1 (1.5%)	3 (1.4%)	1.0
Pocket hematoma^a^	2 (0.7%)	1 (1.5%)	1 (0.5%)	.4
Lead-related adverse events	12 (4.2%)	5 (7.4%)	7 (3.2%)	.1
Atrial lead dislodgement	4 (1.4%)	2 (2.9%)	2 (0.9%)	.2
LBBAP lead dislodgement	6 (2.1%)	2 (2.9%)	4 (1.8%)	.6
Atrial lead failure	1 (0.3%)	0 (0%)	1 (0.5%)	1.0
LBBAP lead failure	1 (0.7%)	1 (2.9%)	0 (0%)	.2
Clinical adverse events (≤30 days)	2 (0.7%)	2 (2.9%)	0 (0%)	.06
Myocardial infarction	0 (0%)	0 (0%)	0 (0%)	1
Stroke/TIA	0 (0%)	0 (0%)	0 (0%)	1
All-cause death^b^	2 (0.7%)	2 (2.9%)	0 (0%)	.06

*Abbreviations:* DVT, deep vein thrombosis; ECG, electrocardiogram; LBBAP, left bundle branch area pacing; TIA, transient ischemic attack. ^a^No intervention required. ^b^None of the deaths were due to implant-related causes.

## Discussion

In this retrospective review, we analyzed the learning curve, device-related adverse events, and key clinical outcomes for operators in their initial 10 implantation attempts (LBBAP_inexp_) compared to their subsequent attempts (LBBAP_exp_). The main findings of this study were that: (1) there was an increase in procedural success measured by LVAT ≤ 80 ms with increased experience (≥10 cases); (2) fluoroscopy times were lower with increased experience, but the total procedure time remained similar; (3) the number of attempts at LBBAP increased with more experience; and (4) there was no difference in device-related adverse events or 30-day all-cause mortality.

### Learning curve of left bundle branch area pacing

In our evaluation of the learning curve and success rate of LBBAP, we found an improvement in procedural success as determined by LVAT ≤ 80 ms after operators had an experience of ≥10 LBBAP implantations (*P* = .04). In contrast, data from the Multicentre European Left Bundle Branch Area Pacing Outcomes Study (MELOS) indicated a slowly rising learning curve, with gradual improvement in the V6 R-wave peak time and QRSd over the first 110 cases, followed by a plateau.^14^ In a smaller study, Gupta et al. found no difference in the LVAT time between the first 20 and the subsequent 60 cases.^15^ Furthermore, Wang et al. found that, for a single operator, the learning curve for LBBAP stabilized after 150 cases.^12^ The discrepancies across studies may be attributed to heterogeneity in institutional protocols, sample sizes, operator training, study methodologies, and patient population.

In LBBAP, it is important to confirm the recruitment of LBB rather than only capture of the LV septal myocardium. LVAT, measured as the time from the pacing stimulus to the peak of V6, is an indicator of the time taken for depolarization to reach the LV lateral wall. In cases where only LV septal myocardium is captured without recruitment of LBB, this measure will often reflect a delay of approximately 15–25 ms. Thus, LVAT ≤ 80 ms can be used as an objective determination of LBB capture. Jastrzebski et al. determined that the LVAT cutoff of 80 ms was 100% specific for LBB capture in patients with diseased LBB, while a cutoff of 74 ms was 100% specific for LBB capture in normal LBBs.^16^ While this cutoff is specific, variables such as LV size and intrinsic LBB delay likely reduce the sensitivity. Many studies have used this LVAT cutoff, in addition to including narrow paced QRS complexes, in determining LBBAP implantation success.^17–19^ It is noteworthy that we did not find any differences in paced QRSd between the inexperienced and experienced groups.

There was no difference in success as self-determined by the operator. However, self-assessments may be affected by inherent biases originating from common cognitive tools for enhancing positive feedback.^20^ Further, individual operators at our institution did not use any established criteria to determine procedural success, but they would generally aim for shortening of the paced LVAT and narrowing of paced QRS complexes.

Prior studies on LBBAP learning curves reported a decrease in both fluoroscopy times and total procedure times with increased operator experience.^12,21,22^ In our analysis, the procedure time did not decrease significantly as the operators gained experience. This might be attributable to an increase in the number of attempts with an increase in experience, lengthening the overall procedure duration. The increase in the number of attempts with experience might have been driven by greater procedural familiarity, a better ability to recognize poor LBBAP capture, and making a quicker decision to repeat attempts. Furthermore, time constraints associated with a high-throughput electrophysiology laboratory may have contributed to a lack of difference between procedure times, where inexperienced operators may have attempted to optimize procedure duration itself instead of LBB capture. Regardless, a decrease in total fluoroscopy time was noticed with increased operator experience.

There were no significant differences in device-related adverse events between experienced and inexperienced operators in our study. Notably, the procedure-related complication rate was 4.5% in our study. It is important to note that this includes both events in patients undergoing complex lead extractions and self-resolving complications, such as pocket hematomas. This rate is similar to or only marginally higher than those in other studies involving LBBAP and may be accounted for by the comprehensive reporting of all complications in our study.^9,12,17,21,23^ Additionally, lead-related adverse events, ie, atrial or LBBAP lead dislodgement or failure within 30 days, occurred in 4.2%, with the most common adverse event being LBBAP lead dislodgement or failure (2.8%). Our findings suggest that LBBAP exhibits a comparatively favorable safety profile in terms of major complications, even in instances where operators lack experience.

### Left bundle branch area pacing and His-bundle pacing

In comparison with HBP, LBBAP in general has more favorable electrical parameters and simplified programming, making the LBBAP procedure easier for operators. Multiple studies have determined that LBBAP has a higher implant success rate and improved pacing parameters compared to HBP.^10,24^ O’Connor et al. also found that the learning curve for LBBAP is shorter than the learning curve for HBP.^13^ This is likely due to the difficulty in locating the His bundle in patients with anatomical variations or an enlarged atrium. As LBBAP leads can be implanted in a relatively larger area of the myocardium, its conduct can be easier for novice operators. Therefore, LBBAP can be considered as an important alternative to HBP, especially for novice operators.

### Limitations

Our study has several limitations. First, this was a retrospective analysis, introducing possibilities for biases and erroneous information. There are limitations in the generalizability of our findings, given that it was conducted at a single tertiary academic center with a predominantly elderly, Caucasian patient population. This study was conducted over the course of 3 years, so changes in implantation techniques and technology over time may have impacted study data. Further, the operators did not receive any formal training for LBBAP. Newer operators likely received informal training from colleagues with more experience in this technique.

## Conclusion

With continuing advancements in conduction system pacing, including LBBAP, these methods are likely to gain widespread adoption. The learning curve for LBBAP is an important consideration when shifting to this procedure. In this study, operators ultimately used less fluoroscopy time and had a higher number of LBBAP implant attempts after their first 10 cases. Furthermore, despite an increase in the success rate with more operator experience, operators without LBBAP experience had relatively high success rates to start with, indicating a manageable learning curve of this novel implant method. It is reassuring that novice operators did not have increased procedure times and, in general, did not have unfavorable success or adverse event rates. This should encourage cardiac electronic implantable device implanters to switch from conventional RVP to LBBAP.
